# Supplementary material for: Discovery and application of insertion-deletion (INDEL) polymorphisms for QTL mapping of early life-history traits in Atlantic salmon
Source: BMC Genomics. 2010 Mar 8;11:156. doi: 10.1186/1471-2164-11-156 (PMC2838853; doi:10.1186/1471-2164-11-156)
Supplement: Additional file 2 — Information on developed 76 locus single-run INDEL panel in Atlantic salmon. Information on fluorescence labeling, primer concentrations, PCR pooling and links to alignments, INDEL motifs and GENESCAN (Burge and Karlin 1997) predictions of genes/exons are available in html format. [file 1471-2164-11-156-S2.ZIP › Additionalfile2/table7157.html]

Contig Alignment

```
Base	A B C D E F G H I J K L M N O P Q R S T U V W X Y Z   
1	. . . . . . . . . . G . . . . . . . . . . . . . . .    
2	. . . . . . . . . . T . . . . . . . . . . . . . . .    
3	. . . . . . . . . . A . . . . . . . . . . . . . . .    
4	. . . . . . . . . . T . . . . . . . . . . . . . . .    
5	. . . G . . . . . . G . . . . . . . . . . . . . . .    
6	. . . C . . . . . . C . . . . . . . . . . . . . . .    
7	. . . A . . . . . . A . . . . . . . . . . . . . . .    
8	. . . G . . . . . . G . . . . . . . . . . . . . . .    
9	. . . C . . . . . . C . . . . . . . . . . . . . . .    
10	. . . T . . . . . . T . . . . . . . . . . . . . . .    
11	. . . T . . . . . . T . . . . . . . . . . . . . . .    
12	. . . T . . . . . . T . . . . . . . . . . . . . . .    
13	. . . A . . . . . . A . . . . . . . . . . . . . . .    
14	. . . T . . . . . . T . . . . . . . . . . . . . . .    
15	. . . T . . . . . . T . . . . . . . . . . . . . . .    
16	. . . A . . . . . . A . . . . . . . . . . . . . . .    
17	. . . A . . . . . . A . . . . . . . . . . . . . . .    
18	. . . C . . . . . . C . . . . . . . . . . . . . . .    
19	. . . T . . . . . . T . . . . . . . . . . . . . . .    
20	. . . A . . . . . . A . . . . . . . . . . . . . . .    
21	. . . A . . . . . . A . . . . . . . . . . . . . . .    
22	. . . A A . . . . A A A . . . . . . . . . . . . . .    
23	. . . T T . . . . T T T . . . . . . . . . . . . . .    
24	. . . G G . . . . G G G . . . . . . . . . . . . . .    
25	. . . A A . . . . A A A . . . . . . . . . . . . . .    
26	. . . T T . . . . T T T . . . . . . . . . . . . . .    
27	. . . T T . . . . T T T . . . . . . . . . . . . . .    
28	. . . T T . . . . T T T . . . . . . . . . . . . . .    
29	. . . T T . . . . T T T . . . . . . . . . . . . . .    
30	. . . A A . . . . A A A . . . . . . . . . . . . . .    
31	. . . A A . . . . A A A . . . . . . . . . . . . . .    
32	. . . T T . . . . T T T . . . . . . . . . . . . . .    
33	. . . G G . . . . G G G . . . . . . . . . . . . . .    
34	. . . A A . . . . A A A . . . . . . . . . . . . . .    
35	. . . A A . . . . A A A . . . . . . . . . . . . . .    
36	. . . A A . . . . A A A . . . . . . . . . . . . . .    
37	. . . T T . . . . T T T . . . . . . . . . . . . . .    
38	. . . T T T . . . T T T . . . . . . . . . . . . . .    
39	. . . A A A . . A A A A . . . . . . . . . . . . . .    
40	. . . C C C . . C C C C . . . . . . . . . . . . . .    
41	. . . A A A . . A A A A . . . . . . . . . . . . . .    
42	. . . T T T . . T T T T T . . . . . . . . . . . . .    
43	. . . T T T . . T T T T T . . . . . . . . . . . . .    
44	. . . G G G . . G G G G G . . . . . . . . . . . . .    
45	. . . T T T . . T T T T T . . . . . . . . . . . . .    
46	. . . T T T . . T T T T T . . . . . . . . . . . . .    
47	. . . T T T . . T T T T T . . . . . . . . . . . . .    
48	. . . G G G . . G G G G G . . . . . . . . . . . . .    
49	. . . C C C . . C C C C C . . . . . . . . . . . . .    
50	. . . A A A . . A A A A A . . . . . . . . . . . . .    
51	. . . A A A . . A A A A A . . . . . . . . . . . . .    
52	. . . A A A . . A A A A A . . . . . . . . . . . . .    
53	. . . C C C . . C C C C C . . . . . . . . . . . . .    
54	. . . T T T . . T T T T T . . . . . . . . . . . . .    
55	. . . G G G . . G G G G G . . . . . . . . . . . . .    
56	. . . A A A . . A A A A A . . . . . . . . . . . . .    
57	. . . T T T . . T T T T T . . . . . . . . . . . . .    
58	. . . A A A . . A A A A A . . . . . . . . . . . . .    
59	. . . T T T . . T T T T T . . . . . . . . . . . . .    
60	. . . G G G . . G G G G G . . . . . . . . . . . . .    
61	. . . T T T . . T T T T T . . . . . . . . . . . . .    
62	. . . G G G . . G G G G G . . . . . . . . . . . . .    
63	. . . A A A . . A A A A A . . . . . . . . . . . . .    
64	. . . C C C . . C C C C C . . . . . . . . . . . . .    
65	. . . A A A . . A A A A A . . . . . . . . . . . . .    
66	. . . C C C . . C C C C C . . . . . . . . . . . . .    
67	. . . A A A . . A A A A A . . . . . . . . . . . . .    
68	. A . A A A A . A A A A A . . . . . . . . . . . . .    
69	. T . T T T T . T T T T T . . . . . . . . . . . . .    
70	. G . G G G G . G G G G G . . . . . . . . . . . . .    
71	. C . C C C C . C C C C C . . . . . . . . . . . . .    
72	. C . C C C C . C C C C C . . . . . . . . . . . . .    
73	. A . A A A A . A A A A A . . . . . . . . . . . . .    
74	. A . A A A A . A A A A A . . . . . . . . . . . . .    
75	. A . A A A A . A A A A A . . . . . . . . . . . . .    
76	. A . A A A A . A A A A A . . . . . . . . . . . . .    
77	. T . T T T T . T T T T T . . . . . . . . . . . . .    
78	. A . A A A A . A A A A A . . . . . . . . . . . . .    
79	A A . A A A A . A A A A A . . . . . . . . . . . . .    
80	C C . C C C C . C C C C C . . . . . . . . . . . . .    
81	A A . A A A A . A A A A A . . . . . . . . . . . . .    
82	G G . A G G G . G G A G G . . . . . . . . . . . . .    
83	G G . A G G G . G G A G G . . . . . . . . . . . . .    
84	C C . C C C C . C C C C C . . . . . . . . . . . . .    
85	A A . A A A A . A A A A A . . . . . . . . . . . . .    
86	A A . A A A A . A A A A A . . . . . . . . . . . . .    
87	A A . A A A A . A A A A A . . . . . . . . . . . . .    
88	A A . A A A A . A A A A A . . . . . . . . . . . . .    
89	C C . C C C C . C C C C C . . . . . . . . . . . . .    
90	T T . T T T T . T T T T T . . . . . . . . . . . . .    
91	A A . A A A A . A A A A A . . . . . . . . . . . . .    
92	T T . T T T T T T T T T T . . . . . . . . . . . . .    
93	T T . T T T T T T T T T T . . . . . . . . . . . . .    
94	T T . T T T T T T T T T T . . . . . . . . . . . . .    
95	T T . T T T T T T T T T T . . . . . . . . . . . . .    
96	T T . T T T T T T T T T T . . . . . . . . . . . . .    
97	T T . T T T T T T T T T T . . . . . . . . . . . . .    
98	G G . G G G G G G G G G G . . . . . . . . . . . . .    
99	C C . C C C C C C C C C C . . . . . . . . . . . . .    
100	T T . T T T T T T T T T T . . . . . . . . . . . . .    
101	A A . A A A A A A A A A A . . . . . . . . . . . . .    
102	A A . A A A A A A A A A A . . . . . . . . . . . . .    
103	A A . A A A A A A A A A A A . . . . . . . . . . . .    
104	C C . C C C C C C C C C C C . . . . . . . . . . . .    
105	A A . A A A A A A A A A A A . . . . . . . . . . . .    
106	G G . G G G G G G G G G G G . . . . . . . . . . . .    
107	A A . A A A A A A A A A A A . . . . . . . . . . . .    
108	G G . G G G G G G G G G G G . . . . . . . . . . . .    
109	C C C C C C C C C C C C C C . . . . . . . . . . . .    
110	C C C C C C C C C C C C C C . . . . . . . . . . . .    
111	C C C C C C C C C C C C C C . . . . . . . . . . . .    
112	C C C C C C C C C C C C C C . . . . . . . . . . . .    
113	A A A A A A A A A A A A A A . . . . . . . . . . . .    
114	C C C C C C C C C C C C C C . . . . . . . . . . . .    
115	C C C C C C C C C C C C C C . . . . . . . . . . . .    
116	T T T T T T T T T T T T T T . . . . . . . . . . . .    
117	G G G G G G G G G G G G G G . . . . . . . . . . . .    
118	A A A A A A A A A A A A A A . . . . . . . . . . . .    
119	T T T T T T T T T T T T T T . . . . . . . . . . . .    
120	T T T T T T T T T T T T T T . . . . . . . . . . . .    
121	G G G G G G G G G G G G G G . . . . . . . . . . . .    
122	T T T T T T T T T T T T T T . . . . . . . . . . . .    
123	G G G G G G G G G G G G G G . . . . . . . . . . . .    
124	T T T T T T T T T T T T T T . . . . . . . . . . . .    
125	G G G G G G G G G G G G G G . . . . . . . . . . . .    
126	A A A A A A A A A A A A A A . . . . . . . . . . . .    
127	A A A A A A A A A A A A A A . . . . . . . . . . . .    
128	A A A A A A A A A A A A A A . . . . . . . . . . . .    
129	A A A A A A A A A A A A A A . . . . . . . . . . . .    
130	T T T T T T T T T T T T T T . . . . . . . . . . . .    
131	A A A A A A A A A A A A A A . . . . . . . . . . . .    
132	A A A A A A A A A A A A A A . . . . . . . . . . . .    
133	G G G G G G G G G G G G G G . . . . . . . . . . . .    
134	T T T T T T T T T T T T T T . . . . . . . . . . . .    
135	T T T T T T T T T T T T T T . . . . . . . . . . . .    
136	C C C C C C C C C C C C C C . . . . . . . . . . . .    
137	A A A A A A A A A A A A A A . . . . . . . . . . . .    
138	G G G G G G G G G G G G G G . . . . . . . . . . . .    
139	C C C C C C C C C C C C C C . . . . . . . . . . . .    
140	T T T T T T T T T T T T T T . . . . . . . . . . . .    
141	T T T T T T T T T T T T T T . . . . . . . . . . . .    
142	G G G G G G G G G G G G G G . . . . . . . . . . . .    
143	T T T T T T T T T T T T T T . . . . . . . . . . . .    
144	A A A A A A A A A A A A A A . . . . . . . . . . . .    
145	A A A A A A A A A A A A A A . . . . . . . . . . . .    
146	A A A A A A A A A A A A A A . . . . . . . . . . . .    
147	A A A A A A A A A A A A A A . . . . . . . . . . . .    
148	T T T T T T T T T T T T T T . . . . . . . . . . . .    
149	G G G G G G G G G G G G G G . . . . . . . . . . . .    
150	T T T T T T T T T T T T T T . . . . . . . . . . . .    
151	T T T T T T T T T T T T T T . . . . . . . . . . . .    
152	G G G G G G G G G G G G G G . . . . . . . . . . . .    
153	A A A A A A A A A A A A A A . . . . . . . . . . . .    
154	A A A A A A A A A A A A A A . . . . . . . . . . . .    
155	T T T T T T T T T T T T T T . . . . . . . . . . . .    
156	A A A A A A A A A A A A A A . . . . . . . . . . . .    
157	A A A A A A A A A A A A A A . . . . . . . . . . . .    
158	A A A A A A A A A A A A A A . . . . . . . . . . . .    
159	T T T T T T T T T T T T T T . . . . . . . . . . . .    
160	A A A A A A A A A A A A A A . . . . . . . . . . . .    
161	C C C C C C C C C C C C C C . . . . . . . . . . . .    
162	A A A A A A A A A A A A A A . . . . . . . . . . . .    
163	A A A A A A A A A A A A A A . . . . . . . . . . . .    
164	T T T T T T T T T T T T T T . . . . . . . . . . . .    
165	A A A A A A A A A A A A A A . . . . . . . . . . . .    
166	T T T T T T T T T T T T T T . . . . . . . . . . . .    
167	C C C C C C C C C C C C C C . . . . . . . . . . . .    
168	A A A A A A A A A A A A A A . . . . . . . . . . . .    
169	T T T T T T T T T T T T T T . . . . . . . . . . . .    
170	A A A A A A A A A A A A A A . . . . . . . . . . . .    
171	C C C C C C C C C C C C C C . . . . . . . . . . . .    
172	A A A A A A A A A A A A A A . . . . . . . . . . . .    
173	T T T T T T T T T T T T T T . . . . . . . . . . . .    
174	A A A A A A A A A A A A A A . . . . . . . . . . . .    
175	G G G G G G G G G G G G G G . . . . . . . . . . . .    
176	G G G G G G G G G G G G G G . . . . . . . . . . . .    
177	T T T T T T T T T T T T T T . . . . . . . . . . . .    
178	G G G G G G G G G G G G G G . . . . . . . . . . . .    
179	C C C C C C C C C C C C C C . . . . . . . . . . . .    
180	T T T T T T T T T T T T T T . . . . . . . . . . . .    
181	A A A A A A A A A A A A A A . . . . . . . . . . . .    
182	A A A A A A A A A A A A A A . . . . . . . . . . . .    
183	A A A A A A A A A A A A A A . . . . . . . . . . . .    
184	A A A A A A A A A A A A A A . . . . . . . . . . . .    
185	A A A A A A A A A A A A A A . . . . . . . . . . . .    
186	C C C C C C C C C C C C C C . . . . . . . . . . . .    
187	A A A A A A A A A A A A A A . . . . . . . . . . . .    
188	T T T T T T T T T T T T T T . . . . . . . . . . . .    
189	G G G G G G G G G G G G G G . . . . . . . . . . . .    
190	G G G G G G G G G G G G G G . . . . . . . . . . . .    
191	T T T T T T T T T T T T T T . . . . . . . . . . . .    
192	T T T T T T T T T T T T T T . . . . . . . . . . . .    
193	A A A A A A A A A A A A A A . . . . . . . . . . . .    
194	A A A A A A A A A A A A A A . . . . . . . . . . . .    
195	T T T T T T T T T T T T T T . . . . . . . . . . . .    
196	T T T T T T T T T T T T T T . . . . . . . . . . . .    
197	C C C C C C C C C C C C C C . . . . . . . . . . . .    
198	C C C C C C C C C C C C C C . . . . . . . . . . . .    
199	A A A A A A A A A A A A A A . . . . . . . . . . . .    
200	T T T T T T T T T T T T T T . . . . . . . . . . . .    
201	T T T T T T T T T T T T T T . . . . . . . . . . . .    
202	G G G G G G G G G G G G G G . . . . . . . . . . . .    
203	A A A A A A A A A A A A A A . . . . . . . . . . . .    
204	C C C C C C C C C C C C C C . . . . . . . . . . . .    
205	C C C C C C C C C C C C C C . . . . . . . . . . . .    
206	T T T T T T T T T T T T T T . . . . . . . . . . . .    
207	G G G G G G G G G G G G G G . . . . . . . . . . . .    
208	A A A A A A A A A A A A A A . . . . . . . . . . . .    
209	A A A A A A A A A A A A A A . . . . . . . . . . . .    
210	A A A A A A A A A A A A A A . . . . . . . . . . . .    
211	A A A A A A A A A A A A A A . . . . . . . . . . . .    
212	A A A A A A A A A A A A A A . . . . . . . . . . . .    
213	C C C C C C C C C C C C C C . . . . . . . . . . . .    
214	A A A A A A A A A A A A A A . . . . . . . . . . . .    
215	C C C C C C C C C C C C C C . . . . . . . . . . . .    
216	T T T T T T T T T T T T T T . . . . . . . . . . . .    
217	A A A A A A A A A A A A A A . . . . . . . . . . . .    
218	T T T T T T T T T T T T T T . . . . . . . . . . . .    
219	T T T T T T T T T T T T T T . . . . . . . . . . . .    
220	A A A A A A A A A A A A A A . . . . . . . . . . . .    
221	G G G G G G G G G G G G G G . . . . . . . . . . . .    
222	A A A A A A A A A A A A A A . . . . . . . . . . . .    
223	G G G G G G G G G G G G G G . . . . . . . . . . . .    
224	T T T T T T T T T T T T T T . . . . . . . . . . . .    
225	G G G G G G G G G G G G G G . . . . . . . . . . . .    
226	A A A A A A A A A A A A A A . . . . . . . . . . . .    
227	G G G G G G G G G G G G G G . . . . . . . . . . . .    
228	A A A A A A A A A A A A A A . . . . . . . . . . . .    
229	T T T T T T T T T T T T T T . . . . . . . . . . . .    
230	T T T T T T T T T T T T T T . . . . . . . . . . . .    
231	G G G G G G G G G G G G G G . . . . . . . . . . . .    
232	G G G G G G G G G G G G G G . . . . . . . . . . . .    
233	A A A A A A A A A A A A A A . . . . . . . . . . . .    
234	T T T T T T T T T T T T T T . . . . . . . . . . . .    
235	A A A A A A A A A A A A A A . . . . . . . . . . . .    
236	A A A A A A A A A A A A A A . . . . . . . . . . . .    
237	A A A A A A A A A A A A A A . . . . . . . . . . . .    
238	C C C C C C C C C C C C C C . . . . . . . . . . . .    
239	C C C C C C C C C C C C C C . . . . . . . . . . . .    
240	C C C C C C C C C C C C C C . . . . . . . . . . . .    
241	A A A A A A A A A A A A A A . . . . . . . . . . . .    
242	C C C C C C C C C C C C C C . . . . . . . . . . . .    
243	A A A A A A A A A A A A A A . . . . . . . . . . . .    
244	A A A A A A A A A A A A A A . . . . . . . . . . . .    
245	A A A A A A A A A A A A A A . . . . . . . . . . . .    
246	A A A A A A A A A A A A A A . . . . . . . . . . . .    
247	A A A A A A A A A A A A A A . . . . . . . . . . . .    
248	T T T T T T T T T T T T T T . . . . . . . . . . . .    
249	A A A A A A A A A A A A A A . . . . . . . . . . . .    
250	C C C C C C C C C C C C C C . . . . . . . . . . . .    
251	A - - A - - - - - A A A A - . . . . . . . . . . . .  6	 indel  
252	C - - C - - - - - C C C C - . . . . . . . . . . . .  6	 indel  
253	C C C C C C C C C C C C C C . . . . . . . . . . . .    
254	T T T T T T T T T T T T T T . . . . . . . . . . . .    
255	G G G G G G G G G G G G G G . . . . . . . . . . . .    
256	G G G G G G G G G G G G G G . . . . . . . . . . . .    
257	G G G G G G G G G G G G G G . . . . . . . . . . . .    
258	T T T T T T T T T T T T T T . . . . . . . . . . . .    
259	T T T T T T T T T T T T T T . . . . . . . . . . . .    
260	T T T T T T T T T T T T T T . . . . . . . . . . . .    
261	A A A A A A A A A A A A A A . . . . . . . . . . . .    
262	C C C C C C C C C C C C C C . . . . . . . . . . . .    
263	A A A A A A A A A A A A A A . . . . . . . . . . . .    
264	C C C C C C C C C C C C C C . . . . . . . . . . . .    
265	A A A A A A A A A A A A A A . . . . . . . . . . . .    
266	T T T T T T T T T T T T T T . . . . . . . . . . . .    
267	T T T T T T T T T T T T T T . . . . . . . . . . . .    
268	T T T T T T T T T T T T T T . . . . . . . . . . . .    
269	T T T T T T T T T T T T T T . . . . . . . . . . . .    
270	T T T T T T T T T T T T T T . . . . . . . . . . . .    
271	G G G G G G G G G G G G G G . . . . . . . . . . . .    
272	T T T T T T T T T T T T T T . . . . . . . . . . . .    
273	A A A A A A A A A A A A A A . . . . . . . . . . . .    
274	C C C C C C C C C C C C C C . . . . . . . . . . . .    
275	C C C C C C C C C C C C C C . . . . . . . . . . . .    
276	A A A A A A A A A A A A A A . . . . . . . . . . . .    
277	A A A A A A A A A A A A A A . . . . . . . . . . . .    
278	A A A A A A A A A A A A A A . . . . . . . . . . . .    
279	A A A A A A A A A A A A A A . . . . . . . . . . . .    
280	T T T T T T T T T T T T T T . . . . . . . . . . . .    
281	C C C C C C C C C C C C C C . . . . . . . . . . . .    
282	A A A A A A A A A A A A A A . . . . . . . . . . . .    
283	A A A A A A A A A A A A A A . . . . . . . . . . . .    
284	C C C C C C C C C C C C C C . . . . . . . . . . . .    
285	A A A A A A A A A A A A A A . . . . . . . . . . . .    
286	T T T T T T T T T T T T T T . . . . . . . . . . . .    
287	T T T T T T T T T T T T T T . . . . . . . . . . . .    
288	C C C C C C C C C C C C C C . . . . . . . . . . . .    
289	C C C C C C C C C C C C C C . . . . . . . . . . . .    
290	T T T T T T T T T T T T T T . . . . . . . . . . . .    
291	G G G G G G G G G G G G G G . . . . . . . . . . . .    
292	A A A A A A A A A A A A A A . . . . . . . . . . . .    
293	A A A A A A A A A A A A A A . . . . . . . . . . . .    
294	T T T T T T T T T T T T T T . . . . . . . . . . . .    
295	T T T T T T T T T T T T T T . . . . . . . . . . . .    
296	T T T T T T T T T T T T T T . . . . . . . . . . . .    
297	T T T T T T T T T T T T T T . . . . . . . . . . . .    
298	C C C C C C C C C C C C C C . . . . . . . . . . . .    
299	C C C C C C C C C C C C C C . . . . . . . . . . . .    
300	T T T T T T T T T T T T T T . . . . . . . . . . . .    
301	G G G G G G G G G G G G G G . . . . . . . . . . . .    
302	T T T T T T T T T T T T T T . . . . . . . . . . . .    
303	G G G G G G G G G G G G G G . . . . . . . . . . . .    
304	A A A A A A A A A A A A A A . . . . . . . . . . . .    
305	T T T T T T T T T T T T T T . . . . . . . . . . . .    
306	G G G G G G G G G G G G G G . . . . . . . . . . . .    
307	T T T T T T T T T T T T T T . . . . . . . . . . . .    
308	T T T T T T T T T T T T T T . . . . . . . . . . . .    
309	G G G G G G G G G G G G G G . . . . . . . . . . . .    
310	T T T T T T T T T T T T T T . . . . . . . . . . . .    
311	C C C C C C C C C C C C C C . . . . . . . . . . . .    
312	C C C C C C C C C C C C C C . . . . . . . . . . . .    
313	T T T T T T T T T T T T T T . . . . . . . . . . . .    
314	T T T T T T T T T T T T T T . . . . . . . . . . . .    
315	T T T T T T T T T T T T T T . . . . . . . . . . . .    
316	C C C C C C C C C C C C C C . . . . . . . . . . . .    
317	C C C C C C C C C C C C C C . . . . . . . . . . . .    
318	A A A A A A A A A A A A A A . . . . . . . . . . . .    
319	A A A A A A A A A A A A A A . . . . . . . . . . . .    
320	T T T T T T T T T T T T T T . . . . . . . . . . . .    
321	G G G G G G G G G G G G G G . . . . . . . . . . . .    
322	T T T T T T T T T T T T T T . . . . . . . . . . . .    
323	A A A A A A A A A A A A A A . . . . . . . . . . . .    
324	T T T T T T T T T T T T T T . . . . . . . . . . . .    
325	A A A A A A A A A A A A A A . . . . . . . . . . . .    
326	C C C C C C C C C C C C C C . . . . . . . . . . . .    
327	A A A A A A A A A A A A A A . . . . . . . . . . . .    
328	G G G G G G G G G G G G G G . . . . . . . . . . . .    
329	T T T T T T T T T T T T T T . . . . . . . . . . . .    
330	G G G G G G G G G G G G G G . . . . . . . . . . . .    
331	C C C C C C C C C C C C C C . . . . . . . . . . . .    
332	A A A A A A A A A A A A A A . . . . . . . . . . . .    
333	T T T T T T T T T T T T T T . . . . . . . . . . . .    
334	T T T T T T T T T T T T T T . . . . . . . . . . . .    
335	T T T T T T T T T T T T T T . . . . . . . . . . . .    
336	A A A A A A A A A A A A A A . . . . . . . . . . . .    
337	A A A A A A A A A A A A A A . . . . . . . . . . . .    
338	C C C C C C C C C C C C C C . . . . . . . . . . . .    
339	C C C C C C C C C C C C C C . . . . . . . . . . . .    
340	C C C C C C C C C C C C C C . . . . . . . . . . . .    
341	T T T T T T T T T T T T T T . . . . . . . . . . . .    
342	C C C C C C C C C C C C C C . . . . . . . . . . . .    
343	A A A A A A A A A A A A A A . . . . . . . . . . . .    
344	T T T T T T T T T T T T T T . . . . . . . . . . . .    
345	T T T T T T T T T T T T T T . . . . . . . . . . . .    
346	C C C C C C C C C C C C C C . . . . . . . . . . . .    
347	C C C C C C C C C C C C C C . . . . . . . . . . . .    
348	C C C C C C C C C C C C C C . . . . . . . . . . . .    
349	A A A A A A A A A A A A A A . . . . . . . . . . . .    
350	C C C C C C C C C C C C C C . . . . . . . . . . . .    
351	A A A A A A A A A A A A A A . . . . . . . . . . . .    
352	G G G G G G G G G G G G G G . . . . . . . . . . . .    
353	A A A A A A A A A A A A A A . . . . . . . . . . . .    
354	T T T T T T T T T T T T T T . . . . . . . . . . . .    
355	A A A A A A A A A A A A A A . . . . . . . . . . . .    
356	A A A A A A A A A A A A A A . . . . . . . . . . . .    
357	G G A G A G G G G G G G G G . . . . . . . . . . . .    
358	A A A A A A A A A A A A A A . . . . . . . . . . . .    
359	A A A A A A A A A A A A A A . . . . . . . . . . . .    
360	C C C C C C C C C C C C C C . . . . . . . . . . . .    
361	A A A A A A A A A A A A A A . . . . . . . . . . . .    
362	G G G G G G G G G G G G G G . . . . . . . . . . . .    
363	C C C C C C C C C C C C C C . . . . . . . . . . . .    
364	C C C C C C C C C C C C C C . . . . . . . . . . . .    
365	G A A G A A A A A A G A A A . . . . . . . . . . . .    
366	G G G G G G G G G G G G G G . . . . . . . . . . . .    
367	A A A A A A A A A A A A A A . . . . . . . . . . . .    
368	C C C C C C C C C C C C C C . . . . . . . . . . . .    
369	T T T T T T T T T T T T T T . . . . . . . . . . . .    
370	C C C C C C C C C C C C C C . . . . . . . . . . . .    
371	T T T T T T T T T T T T T T . . . . . . . . . . . .    
372	G G G G G G G G G G G G G G . . . . . . . . . . . .    
373	T T T T T T T T T T T T T T . . . . . . . . . . . .    
374	A A A A A A A A A A A A A A . . . . . . . . . . . .    
375	A A A A A A A A A A A A A A . . . . . . . . . . . .    
376	A A A A A A A A A A A A A A . . . . . . . . . . . .    
377	C C C C C C C C C C C C C C . . . . . . . . . . . .    
378	C C C C C C C C C C C C C C . . . . . . . . . . . .    
379	C C C C C C C C C C C C C C . . . . . . . . . . . .    
380	C C C C C C C C C C C C C C . . . . . . . . . . . .    
381	T T T T T T T T T T T T T T . . . . . . . . . . . .    
382	C C C C C C C C C C C C C C . . . . . . . . . . . .    
383	C C C C C C C C C C C C C C . . . . . . . . . . . .    
384	A A A A A A A A A A A A A A . . . . . . . . . . . .    
385	G G G G G G G G G G G G G G . . . . . . . . . . . .    
386	A A A A A A A A A A A A A A . . . . . . . . . . . .    
387	A A A A A A A A A A A A A A . . . . . . . . . . . .    
388	T T T T T T T T T T T T T T . . . . . . . . . . . .    
389	A A A A A A A A A A A A A A . . . . . . . . . . . .    
390	C C C C C C C C C C C C C C . . . . . . . . . . . .    
391	A A A A A A A A A A G A A A . . . . . . . . . . . .    
392	C C C C C C C C C C C C C C . . . . . . . . . . . .    
393	A A A A A A A A A A A A A A . . . . . . . . . . . .    
394	C C C C C C C C C C C C C C . . . . . . . . . . . .    
395	A A A A A A A A A A A A A A . . . . . . . . . . . .    
396	T T T T T T T T T T T T T T . . . . . . . . . . . .    
397	T T T T T T T T T T T T T T . . . . . . . . . . . .    
398	A A A A A A A A A A A A A A . . . . . . . . . . . .    
399	C C C C C C C C C C C C C C . . . . . . . . . . . .    
400	T T T T T T T T T T T T T T . . . . . . . . . . . .    
401	C C C C C C C C C C C C C C . . . . . . . . . . . .    
402	A A A A A A A A A A A A A A . . . . . . . . . . . .    
403	A A A A A A A A A A A A A A . . . . . . . . . . . .    
404	A A A A A A A A A A A A A A . . . . . . . . . . . .    
405	A A A A A A A A A A A A A A . . . . . . . . . . . .    
406	A A A A A A A A A A A A A A . . . . . . . . . . . .    
407	C C C C C C C C C C C C C C . . . . . . . . . . . .    
408	C C C C C C C C C C C C C C . . . . . . . . . . . .    
409	T T T T T T T T T T T T T T . . . . . . . . . . . .    
410	A A A A A A A A A A A A A A . . . . . . . . . . . .    
411	A A A A A A A A A A A A A A . . . . . . . . . . . .    
412	T T T T T T T T T T T T T T . . . . . . . . . . . .    
413	C C C C C C C C C C C C C C . . . . . . . . . . . .    
414	C C C C C C C C C C C C C C . . . . . . . . . . . .    
415	T T T T T T T T T T T T T T . . . . . . . . . . . .    
416	C C C C C C C C C C C C C C . . . . . . . . . . . .    
417	A A A A A A A A A A A A A A . . . . . . . . . . . .    
418	C C C C C C C C C C C C C C . . . . . . . . . . . .    
419	C C C C C C C C C C C C C C . . . . . . . . . . . .    
420	A A A A A A A A A A A A A A . . . . . . . . . . . .    
421	C C C C C C C C C C C C C C . . . . . . . . . . . .    
422	G G G G G G G G G G G G G G . . . . . . . . . . . .    
423	G G G G G G G G G G G G G G . . . . . . . . . . . .    
424	A A A A A A A A A A A A A A . . . . . . . . . . . .    
425	T T T T T T T T T T T T T T . . . . . . . . . . . .    
426	G G G G G G G G G G G G G G . . . . . . . . . . . .    
427	G G G G G G G G G G G G G G . . . . . . . . . . . .    
428	A A A A A A A A A A A A A A . . . . . . . . . . . .    
429	C C C C C C C C C C C C C C . . . . . . . . . . . .    
430	C C C C C C C C C C C C C C . . . . . . . . . . . .    
431	T T T T T T T T T T T T T T . . . . . . . . . . . .    
432	T T T T T T T T T T T T T T . . . . . . . . . . . .    
433	C C C C C C C C C C C C C C . . . . . . . . . . . .    
434	T T T T T T T T T T T T T T . . . . . . . . . . . .    
435	T T T T T T T T T T T T T T . . . . . . . . . . . .    
436	G G G G G G G G G G G G G G . . . . . . . . . . . .    
437	T T T T T T T T T T T T T T . . . . . . . . . . . .    
438	C C C C C C C C C C C C C C . . . . . . . . . . . .    
439	C C C C C C C C C C C C C C . . . . . . . . . . . .    
440	A A A A A A A A A A A A A A . . . . . . . . . . . .    
441	T T T T T T T T T T T T T T . . . . . . . . . . . .    
442	C C C C C C C C C C C C C C . . . . . . . . . . . .    
443	A A A A A A A A A A A A A A . . . . . . . . . . . .    
444	C C C C C C C C C C C C C C . . . . . . . . . . . .    
445	A A A A A A A A A A A A A A . . . . . . . . . . . .    
446	A A A A A A A A A A A A A A . . . . . . . . . . . .    
447	A A A A A A A A A A A A A A . . . . . . . . . . . .    
448	C C C C C C C C C C C C C C . . . . . . . . . . . .    
449	T T T T T T T T T T T T T T . . . . . . . . . . . .    
450	G G G G G G G G G G G G G G . . . . . . . . . . . .    
451	T T T T T T T T T T T T T T . . . . . . . . . . . .    
452	C C C C C C C C C C C C C C . . . . . . . . . . . .    
453	C C C C C C C C C C C C C C . . . . . . . . . . . .    
454	C C C C C C C C C C C C C C . . . . . . . . . . . .    
455	T T T T T T T T T T T T T T . . . . . . . . . . . .    
456	G G G G G G G G G G G G G G . . . . . . . . . . . .    
457	C C C C C C C C C C C C C C . . . . . . . . . . . .    
458	G G G G G G G G G G G G G G . . . . . . . . . . . .    
459	G G G G G G G G G G G G G G . . . . . . . . . . . .    
460	C C C C C C C C C C C C C C . . . . . . . . . . . .    
461	T T T T T T T T T T T T T T . . . . . . . . . . . .    
462	C C C C C C C C C C C C C C . . . . . . . . . . . .    
463	C C C C C C C C C C C C C C . . . . . . . . . . . .    
464	C C C C C C C C C C C C C C . . . . . . . . . . . .    
465	C C C C C C C C C C C C C C . . . . . . . . . . . .    
466	A A A A A A A A A A A A A A . . . . . . . . . . . .    
467	G G G G G G G G G G G G G G . . . . . . . . . . . .    
468	C C C C C C C C C C C C C C . . . . . . . . . . . .    
469	C C C C C C C C C C C C C C . . . . . . . . . . . .    
470	C C C C C C C C C C C C C C . . . . . . . . . . . .    
471	C C C C C C C C C C C C C C . . . . . . . . . . . .    
472	C C C C C C C C C C C C C C . . . . . . . . . . . .    
473	G G G G G G G G G G G G G G . . . . . . . . . . . .    
474	C C C C C C C C C C C C C C . . . . . . . . . . . .    
475	A A A A A A A A A A A A A A . . . . . . . . . . . .    
476	G G G G G G G G G G G G G G . . . . . . . . . . . .    
477	G G G G G G G G G G G G G G . . . . . . . . . . . .    
478	A A A A A A A A A A A A A A . . . . . . . . . . . .    
479	A A A A A A A A A A A A A A . . . . . . . . . . . .    
480	G G G G G G G G G G G G G G . . . . . . . . . . . .    
481	C C C C C C C C C C C C C C . . . . . . . . . . . .    
482	A A A A A A A A A A A A A A . . . . . . . . . . . .    
483	G G G G G G G G G G G G G G . . . . . . . . . . . .    
484	G G G G G G G G G G G G G G . . . . . . . . . . . .    
485	G G G G G G G G G G G G G G . . . . . . . . . . . .    
486	C C C C C C C C C C C C C C . . . . . . . . . . . .    
487	C C C C C C C C C C C C C C . . . . . . . . . . . .    
488	A A A A A A A A A A A A A A . . . . . . . . . . . .    
489	G G G G G G G G G G G G G G . . . . . . . . . . . .    
490	G G G G G G G G G G G G G G . G . . . . . . . . . .    
491	T T T T T T T T T T T T T T . T . . . . . . . . . .    
492	- - - - - - N - - - - - - - . - . . . . . . . . . .    
493	A A A A A A A A A A A A A A . A . . . . . . . . . .    
494	A A A A A A A A A A A A A A . A . . . . . . . . . .    
495	A A A A A A A A A A A A A A . A . . . . . . . . . .    
496	C C C C C C C C C C C C C C . C . . . . . . . . . .    
497	G G G G G G G G G G G G G G . G . . . . . . . . . .    
498	G G G G G G G G G G G G G G . G . . . . . . . . . .    
499	G G G G G G G G G G G G G G . G . . . . . . . . . .    
500	G G G G G G G G G G G G G G . G . . . . . . . . . .    
501	G G G G G G G G G G G G G G . G . . . . . . . . . .    
502	T T T T T T T T T T T T T T . T . . . . . . . . . .    
503	G G G G G G G G G G G G G G . G . . . . . . . . . .    
504	A A A A A A A A A A A A A A . A . . . . . . . . . .    
505	T T T T T T T T T T T T T T . T . . . . . . . . . .    
506	G G G G G G G G G G G G G G . G . . . . . . . . . .    
507	G G G G G G G G G G G G G G . G . . . . . . . . . .    
508	C C C C C C C C C C C C C C . C . . . . . . . . . .    
509	A A A A A A A A A A A A A A . A . . . . . . . . . .    
510	C C C C C C C C C C C C C C . C . . . . . . . . . .    
511	C C C C C C C C C C C C C C . C . . . . . . . . . .    
512	C C C C C C C C C C C C C C . C . . . . . . . . . .    
513	T T T T T T T T T T T T T T T T . . . . . . . . . .    
514	C C C C C C C C C C C C C C C C . . . . . . . . . .    
515	G G G G G G G G G G G G G G G G . . . . . . . . . .    
516	T T T T T T T T T T T T T T T T . . . . . . . . . .    
517	C C C C C C C C C C C C C C C C . . . . . . . . . .    
518	A A A A A A A A A A A A A A A A . . . . . . . . . .    
519	G G G G G G G G G G G G G G G G . . . . . . . . . .    
520	G G G G G G G G G G G G G G G G . . . . . . . . . .    
521	T T T T G T T T T T T T T T T T . . . . . . . . . .    
522	G G G G G G G G G G G G G G G G . . . . . . . . . .    
523	A A A A A A A A A A A A A A A A . . . . . . . . . .    
524	C C C C C C C C C C C C C C C C . . . . . . . . . .    
525	T T T T T T T T T T T T T T T T . . . . . . . . . .    
526	T T T T T T T T T T T T T T T T . . . . . . . . . .    
527	G G G G G G G G G G G G G G G G . . . . . . . . . .    
528	G G G G G G G G G G G G G G G G . . . . . . . . . .    
529	- - - - - - G - - - - - - - - - . . . . . . . . . .    
530	T T T T T T T T T T T T T T T T . . . . . . . . . .    
531	G G G G G G G G G G G G G G G G . . . . . . . . . .    
532	. G G G G G G G G G G G G G G G . . . . . . . . . .    
533	. C C C C C C C C C C C C C C C . . . . . . . . . .    
534	. G G G G G G G G G G G G G G G . . . . . . . . . .    
535	. T T T T T T T T T T T T T T T . . . . . . . . . .    
536	. T T T T T T T T T T T T T T T . . . . . . . . . .    
537	. G G G G G G G G G G G G G G G . . . . . . . . . .    
538	. G G G G G G G G G G G G G G G . . . . . . . . . .    
539	. G G G G G G G G G G G G G G G . . . . . . . . . .    
540	. C C C C C C C C C C C C C C C . . . . . . . . . .    
541	. C C C C C C C C C C C C C C C . . . . . . . . . .    
542	. T C C C C C C C C C C C C C C . . . . . . . . . .    
543	. . A A A A A A A A A A A A A A . . . . . . . . . .    
544	. . G G G G G G G G G G G G G G . . . . . . . . . .    
545	. . C C C C C C C C C C C C C C . . . . . . C . . .    
546	. . C C C C C C C C C C C C C C . . . . . . C . . .    
547	. . A A A A A A A A A A A A A A . . . . . . A . . .    
548	. . T T T T T T T T T T T T T T . . . . . . T . . .    
549	. . A A A A A A A A A A A A A A . . . . . . A . . .    
550	. . T T T T T T T T T T T T T T . . . . . . T . . .    
551	. . C C C C C C C C C C C C C C . . . . . . C . . .    
552	. . G G G G G G G G G G G G G G . . . . . . G . . .    
553	. . G G G G G G G G G G G G G G . . . . . . G . . .    
554	. . T T T T T T T T T T T T T T . . . . . . T . . .    
555	. . C C C C C C C C C C C C C C . . . . . . C . . .    
556	. . C C C C C C C C C C C C C C . . . . . . C . . .    
557	. . T T T T T T T T T T T T T T . . . . . . T . . .    
558	. . C C C C C C C C C C C C C C . . . . . . C . . .    
559	. . A A A A A A A A A A A A A A . . . . A . A . . .    
560	. . C C C C C C C C C C C C C C . . . . C . C . . .    
561	. . C C C C C C C C C C C C C C . . . . C . C . . .    
562	. . C C C C C C C C C C C C C C . . . . C . C . . .    
563	. . A A A A A A A A A A A A A A . . . . A . A . . .    
564	. . G G G G G G G G G G G G G G . . . . G . G . . .    
565	. . C C C C C C C C C C C C C C . . . . C . C . . .    
566	. . C C C C C C C C C C C C C C . . . . C . C . . .    
567	. . C C C C C C C C C C C C C C . . . . C . C . . .    
568	. . G G G G G G G G G G G G G G . . . . G . G . . .    
569	. . G G G G G G G G G G G G G G . . . . G . G . . .    
570	. . G G G G G G G G G G G G G G . . . . G . G . . .    
571	. . C C C C C C C C C C C C C C . . . . C . C . . .    
572	. . A A A A A A A A A A A A A A . . . . A . A . . .    
573	. . G G G G G G G G G G G G G G . . . . G . G . . .    
574	. . C C C C C C C C C C C C C C . . . . C . C . . .    
575	. . A A A A A A A A A A A A A A . . . . A . A . . A    
576	. . C C C C C C C C C C C C C C . . . . C . C . . C    
577	. . G G G G G G G G G G G G G G . . . . G . G . . G    
578	. . C C C C C C C C C C C C C C . . . . C . C . . C    
579	. . A A A A A A A A A A A A A A . . . . A . A . . A    
580	. . T T T T T T T T T T T T T T . . . . T . T . . T    
581	. . T T T T T T T T T T T T T T . . . . T . T . . T    
582	. . C C C C C C C C C C C C C C . . . . C . C . . C    
583	. . . A A A A A A A A A A A A A . . . . A . A . . A    
584	. . . G A G G G G G G G G G G G . . . . G . G . . G    
585	. . . A . A A A A A A A A A A A . . . . A . A . . A    
586	. . . A . A A A A A A A A A A A . . . . A . A . . A    
587	. . . G . A G G G G G G G G G G . . . . G . G . . G    
588	. . . G . . G G G G G G G G G G . . . . G G G . . G    
589	. . . A . . A A A A A A A A A A . . . . A A A . . A    
590	. . . T . . T T T T T T T T T T . . . . T T T . . T    
591	. . . C . . C C C C C C C C C C . . . . C C C . . C    
592	. . . T . . T T T T T T T T T T . . . . T T T . . T    
593	. . . C . . G G G G G G G G G G . . . . G G G . . G    
594	. . . . . . A A A A A A A A A A . . . . A A A . . A    
595	. . . . . . T T T T T T T T T T . . . . T T T . . T    
596	. . . . . . C C C C C C C C C C . . . C C C C . . C    
597	. . . . . . A A A A A A A A A A . . . A A A A . . A    
598	. . . . . . G G G G G G G G G G . . . G G G G . . G    
599	. . . . . . C C C C C C C C C C . . . C C C C . . C    
600	. . . . . . T T T T T T T T T T . . . T T T T . . T    
601	. . . . . . G G G G G G G G G G . . . G G G G . . G    
602	. . . . . . G G G G G G G G G G . . . G G G G . . G    
603	. . . . . . T T T T T T T T T T . . . T T T T . . T    
604	. . . . . . C C C C C C C C C C . . . C C C C . . C    
605	. . . . . . T T T T T T T T T T . . . T T T T . . T    
606	. . . . . . C C C C C C C C C C . . . C C C C . . C    
607	. . . . . . T T T T T T T T T T . . . T T T T . . T    
608	. . . . . . C C C C C C C C C C . . . C C C C . . C    
609	. . . . . . A A A A A A A A A A . . . A A A A . . A    
610	. . . . . . T T T T T T T T T T . . . T T T T . . T    
611	. . . . . . G G G G G G G G G G . . . G G G G . . G    
612	. . . . . . C C C C C C C C C C . . . C C C C . . C    
613	. . . . . . C C C C C C C C C C . . . C C C C . . C    
614	. . . . . . C C C C C C C C C C . . . C C C C . . C    
615	. . . . . . C C C C C C C C C C . . . C C C C . . C    
616	. . . . . . A A A A A A A A A A . . . A A A A . . A    
617	. . . . . . G G G G G G G G G G . . . G G G G . . G    
618	. . . . . . C C C C C C C C C C . . . C C C C . . C    
619	. . . . . . T T T T T T T T T T . . . T T T T . . T    
620	. . . . . . T T T T T T T T T T . . . T T T T . . T    
621	. . . . . . . G G G G G G G G G . . . G G G G . . G    
622	. . . . . . . C C C C C C C C C . . . C C C C . . C    
623	. . . . . . . G G G G G G G G G . . . G G G G . . G    
624	. . . . . . . G G G G G G G G G . . G G G G G . . G    
625	. . . . . . . G G G G G G G G G . . G G G G G . . G    
626	. . . . . . . C C C C C C C C C . . C C C C C . . C    
627	. . . . . . . A A A A A A A A A . . A A A A A . . A    
628	. . . . . . . T T T T T T T T T . . T T T T T . . T    
629	. . . . . . . G G G G G G G G G . . G G G G G . . G    
630	. . . . . . . G G G G G G G G G . . G G G G G . . G    
631	. . . . . . . A A A A A A A A A . . A A A A A . . A    
632	. . . . . . . T T T T T T T T T . . T T T T T . . T    
633	. . . . . . . C C C C C C C C C . . C C C C C . C C    
634	. . . . . . . C C C C C C C C C . . C C C T C . C C    
635	. . . . . . . T T T T T T T T T . . T T T T T . T T    
636	. . . . . . . G G G G G G G G G . . G G G G G . G G    
637	. . . . . . . G G G G G G G G G . . G G G G G . G G    
638	. . . . . . . A A A A A A A A A . . A A A A A . A A    
639	. . . . . . . G G G G G G G G G . . G G G G G . G G    
640	. . . . . . . A A A A A A A A A . . A A A A A . A A    
641	. . . . . . . G G G G G G G G G . . G G G G G . G G    
642	. . . . . . . C C C C C C C C C . . C C C C C . C C    
643	. . . . . . . A A A A A A A A A . . A A A A A . A A    
644	. . . . . . . C C C C C C C C C . . C C C C C . C C    
645	. . . . . . . G G G G G G G G G . . G G G G G . G G    
646	. . . . . . . G G G G G G G G G . . G G G G G . G G    
647	. . . . . . . T T T T T T T T T . . T T T T T . T T    
648	. . . . . . . G G G G G G G G G . . G G G G G . G G    
649	. . . . . . . A A A A A A A A A . . A A A A A . A A    
650	. . . . . . . - - - - - - - - - . . - - - G - . - A    
651	. . . . . . . G G G G G G G G G . . G G G G G . G G    
652	. . . . . . . G G G G G G G G G . . G G G G G . G G    
653	. . . . . . . C C C C C C C C C . . C C C C C . C C    
654	. . . . . . . C C C C C C C C C . . C C C C C . C C    
655	. . . . . . . T T T T T T T T T . . T T T T T . T T    
656	. . . . . . . G G G G G G G G G . . G G G G G . G G    
657	. . . . . . . T T T T T T T T T . . T T T T T . T T    
658	. . . . . . . T T T T T T T T T . . T T T T T . T T    
659	. . . . . . . T T T T T T T T T . . T T T T T . T T    
660	. . . . . . . T T T T T T T T T . . T T T T T . T T    
661	. . . . . . . G G G G G G G G G . . G G G G G . G G    
662	. . . . . . . G G G G G G G G G . . G G G G G . G G    
663	. . . . . . . A A A A A A A A A . . A A A A A . A A    
664	. . . . . . . C C C C C C C C C . . C C C C C . C C    
665	. . . . . . . A A A A A A A A A . . A A A A A . A A    
666	. . . . . . . C C C C C C C C C . . C C C C C . C C    
667	. . . . . . . A A A A A A A A A . . A A A A A . A A    
668	. . . . . . . C C C C C C C C C . . C C C C C . C C    
669	. . . . . . . C C C C C C C C C . . C C C C C . C C    
670	. . . . . . . G G G G G G G G G . . G G G G G . G G    
671	. . . . . . . T T T T T T T T T . . T T T T T . T T    
672	. . . . . . . A A . A A A A A A . . A A A A A . A A    
673	. . . . . . . G G . G G G G G G . . G G G G G . G G    
674	. . . . . . . G G . G G G G G G . . G G G G G . G G    
675	. . . . . . . C C . C C C C C C . . C C C C C . C C    
676	. . . . . . . T T . T T T T T T . . T T T T T . T T    
677	. . . . . . . G G . G G G G G G . . G G G G G . G G    
678	. . . . . . . T T . T T T T T T . . T T T T T . T T    
679	. . . . . . . G G . G G G G G G . . G G G G G . G G    
680	. . . . . . . T T . T T T T T T . . T T T T T . T T    
681	. . . . . . . C C . C C C C C C . . C C C C C . C C    
682	. . . . . . . G G . G G G G G G . . G G G G G . G G    
683	. . . . . . . G G . G G G G G G . . G G G G G . G G    
684	. . . . . . . G . . G G G G G G . . G G G G G . G G    
685	. . . . . . . C . . C C C C C C . . C C C C C . C C    
686	. . . . . . . C . . C C C C C C . . C C C C C . C C    
687	. . . . . . . A . . A A A A A A . . A A A A A . A A    
688	. . . . . . . G . . G G G G G G . . G G G G G . G G    
689	. . . . . . . C . . C C C C C C . . C C C C C . C C    
690	. . . . . . . C . . C C C C C C . . C C C C C . C C    
691	. . . . . . . C . . C C C C C C . . C C C C C . C C    
692	. . . . . . . C . . C C C C C C . . C C C C C . C C    
693	. . . . . . . C . . C C C C C C . . C C C C C . C C    
694	. . . . . . . C . . C C C C C C . . C C C C C . C C    
695	. . . . . . . . . . T T T T T T . . T T T T T . T T    
696	. . . . . . . . . . G G G G G G . . G G G G G . G G    
697	. . . . . . . . . . G G G G G G . . G G G G G . G G    
698	. . . . . . . . . . G G G G G G . . G G G G G . G G    
699	. . . . . . . . . . A A A A A A . . A A A A A . A A    
700	. . . . . . . . . . G G G G G G . . G G G G G . G G    
701	. . . . . . . . . . T T T T T T . . T T T T T . T T    
702	. . . . . . . . . . G G G G G G . . G G G G G . G G    
703	. . . . . . . . . . C C C C C C . . C C C C C . C C    
704	. . . . . . . . . . G G G G G G . . G G G G G . G G    
705	. . . . . . . . . . C C C C C C . . C C C C C . C C    
706	. . . . . . . . . . C C C C C C . . C C C C C . C C    
707	. . . . . . . . . . C C C C C C . . C C C C C . C C    
708	. . . . . . . . . . C C C C C C . . C C C C C . C C    
709	. . . . . . . . . . T T T T T T . . T T T T T . T T    
710	. . . . . . . . . . G G G G G G . . G G G G G . G G    
711	. . . . . . . . . . C C C C C C . . C C C C C . C C    
712	. . . . . . . . . . C C C C C C . . C C C C C . C C    
713	. . . . . . . . . . T T T T T T . . T T T T T . T T    
714	. . . . . . . . . . G G G G G G . . G G G G G . G G    
715	. . . . . . . . . . G G G G G G . . G G G G G . G G    
716	. . . . . . . . . . G G G G G G . . G G G G G . G G    
717	. . . . . . . . . . C C C C C C . . C C C C C . C C    
718	. . . . . . . . . . C C C C C C . . C C C C C . C C    
719	. . . . . . . . . . T T T T T T . . T T T T T . T T    
720	. . . . . . . . . . C C C C C C . . C C C C C . C C    
721	. . . . . . . . . . C C C C C C . . C C C C C . C C    
722	. . . . . . . . . . T T T T T T . . T T T T T . T T    
723	. . . . . . . . . . G G G G G G . . G G G G G . G G    
724	. . . . . . . . . . G G G G G G . . G G G G G . G G    
725	. . . . . . . . . . A A A A A A . . A A A A A . A A    
726	. . . . . . . . . . C C C C C C . . C C C C C . C C    
727	. . . . . . . . . . A A A A A A . . A A A A A . A A    
728	. . . . . . . . . . A A A A A A . . A A A A A . A A    
729	. . . . . . . . . . G A A A A A . . A A A A G . A A    
730	. . . . . . . . . . C T T C T T . . C T C T C . C T  6	 transition  
731	. . . . . . . . . . C C C C C C . . C C C C C . C C    
732	. . . . . . . . . . T T T T T T . . T T T T T . T T    
733	. . . . . . . . . . C C C C C C . . C C C C C . C C    
734	. . . . . . . . . . T T T T T T . . T T T T T . T T    
735	. . . . . . . . . . C . C C C C . . C C C C C . C C    
736	. . . . . . . . . . C . C C C C . . C C C C C . C C    
737	. . . . . . . . . . A . A A A A . . A A A A A . A A    
738	. . . . . . . . . . T . T T T T . . T T T T T . T T    
739	. . . . . . . . . . C . C C C C . . C C C C C . C C    
740	. . . . . . . . . . A . A A A A . . A A A A A . A A    
741	. . . . . . . . . . G . G G G G . . G G G G G . G G    
742	. . . . . . . . . . C . C C C C . . C C C C C . C C    
743	. . . . . . . . . . C . C C C C . . C C C C C . C C    
744	. . . . . . . . . . C . C C C C . . C C C C C . C C    
745	. . . . . . . . . . C . C C C C . . C C C C C . C C    
746	. . . . . . . . . . A . A A A A . . A A A A A . A A    
747	. . . . . . . . . . C . C C C C . . C C C C C . C C    
748	. . . . . . . . . . C . C C C C . . C C C C C . C C    
749	. . . . . . . . . . . . A A A A . . A A A A A . A A    
750	. . . . . . . . . . . . G G G G . . G G G G G . G G    
751	. . . . . . . . . . . . C C C C . . C C C C C . C C    
752	. . . . . . . . . . . . T T T T . . T T T T T . T T    
753	. . . . . . . . . . . . C C C C . . C C C C C . C C    
754	. . . . . . . . . . . . C C C C . . C C C C C . C C    
755	. . . . . . . . . . . . T T T T . . T T T T T . T T    
756	. . . . . . . . . . . . C C C C . . C C C C C . C C    
757	. . . . . . . . . . . . C C C C . . C C C C C . C C    
758	. . . . . . . . . . . . T T T T . . T T T T T . T T    
759	. . . . . . . . . . . . C C C C . . C C C C C . C C    
760	. . . . . . . . . . . . T T T T . . T T T T T . T T    
761	. . . . . . . . . . . . G G G G . . G G G G G . G G    
762	. . . . . . . . . . . . T T T T . . T T T T T T T T    
763	. . . . . . . . . . . . G G G G . . G G G G G G G G    
764	. . . . . . . . . . . . A A A A . . A A A A A A A A    
765	. . . . . . . . . . . . T T T T . . T T T T T T T T    
766	. . . . . . . . . . . . G G G G . . G G G G G G G G    
767	. . . . . . . . . . . . T T T T . . T T T T T T T T    
768	. . . . . . . . . . . . C C C C . . C C C C C C C C    
769	. . . . . . . . . . . . A A A A . . A A A A A A A A    
770	. . . . . . . . . . . . T T T T . . T T T T T T T T    
771	. . . . . . . . . . . . T T T T . . T T T T T T T T    
772	. . . . . . . . . . . . G G G G . . G G G G G G G G    
773	. . . . . . . . . . . . C C C C . . C C C C C C C C    
774	. . . . . . . . . . . . T T T T . . T T T T T T T T    
775	. . . . . . . . . . . . G G G G . . G G G G G G G G    
776	. . . . . . . . . . . . C C C C . . C C C C C C C C    
777	. . . . . . . . . . . . G G G G . . G G G G G G G G    
778	. . . . . . . . . . . . G G G G . . G G G G G G G G    
779	. . . . . . . . . . . . A A A A . . A A A A A A A A    
780	. . . . . . . . . . . . A A A A . . A A A A A A A A    
781	. . . . . . . . . . . . C C C C . . C C C C C C C C    
782	. . . . . . . . . . . . C C C C . . C C C C C C C C    
783	. . . . . . . . . . . . G G G G . . G G G G G G G G    
784	. . . . . . . . . . . . G G G G . . G G G G G G G G    
785	. . . . . . . . . . . . G G G G . . G G G G G G G G    
786	. . . . . . . . . . . . C C C C . . C C C C C C C C    
787	. . . . . . . . . . . . C C C C . . C C C C C C C C    
788	. . . . . . . . . . . . T T T T . . T T T T T T T T    
789	. . . . . . . . . . . . G G G G . . G G G G G G G G    
790	. . . . . . . . . . . . G G G G . . G G G G G G G G    
791	. . . . . . . . . . . . A A A A . . A A A A A A A A    
792	. . . . . . . . . . . . G G G G . . G G G G G G G G    
793	. . . . . . . . . . . . T T T T . . T T T T T T T T    
794	. . . . . . . . . . . . T T T T . . T T T T T T T T    
795	. . . . . . . . . . . . C C C C . . C C C C C C C C    
796	. . . . . . . . . . . . A A A A . . A A A A A A A A    
797	. . . . . . . . . . . . G G G G . . G G G G G G G G    
798	. . . . . . . . . . . . G G G G . . G G G G G G G G    
799	. . . . . . . . . . . . G G G G . . G G G G G G G G    
800	. . . . . . . . . . . . C C C C . . C C C C C C C C    
801	. . . . . . . . . . . . T T T T . . T T T T T T T T    
802	. . . . . . . . . . . . G G G G . . G G G G G G G G    
803	. . . . . . . . . . . . C C C C . . C C C C C C C C    
804	. . . . . . . . . . . . A A A A . . A A A A A A A A    
805	. . . . . . . . . . . . G G G G . . G G G G G G G G    
806	. . . . . . . . . . . . C C C C . . C C C C C C C C    
807	. . . . . . . . . . . . G G G G . . G G G G G G G G    
808	. . . . . . . . . . . . G G G G . . G G G G G G G G    
809	. . . . . . . . . . . . T T T T . . T T T T T T T T    
810	. . . . . . . . . . . . T T T T . . T T T T T T T T    
811	. . . . . . . . . . . . C C C C . . C C C C C C C C    
812	. . . . . . . . . . . . A A A A . . A A A A A A A A    
813	. . . . . . . . . . . . G G G G . . G G G G G G G G    
814	. . . . . . . . . . . . G G G G . . G G G G G G G G    
815	. . . . . . . . . . . . G G G G . . G G G G G G G G    
816	. . . . . . . . . . . . C C C C . . C C C C C C C C    
817	. . . . . . . . . . . . G G G G . . G G G G G G G G    
818	. . . . . . . . . . . . A A A A . . A A A A A A A A    
819	. . . . . . . . . . . . T T T T . . T T T T T T T T    
820	. . . . . . . . . . . . G G G G . . G G G G G G G G    
821	. . . . . . . . . . . . G G G G . . G G G G G G G G    
822	. . . . . . . . . . . . A A A A . . A A A A A A A A    
823	. . . . . . . . . . . . G G G G . . G G G G G G G G    
824	. . . . . . . . . . . . C C C C . . C C C C C C C C    
825	. . . . . . . . . . . . T T T T . . T T T T T T T T    
826	. . . . . . . . . . . . C C C C . . C C C C C C C C    
827	. . . . . . . . . . . . A A A A . . A A A A A A A A    
828	. . . . . . . . . . . . T T T T . . T T T T T T T T    
829	. . . . . . . . . . . . A A A A . . A A A A A A A A    
830	. . . . . . . . . . . . A A A A . . A A A A A A A A    
831	. . . . . . . . . . . . C C C C . . C C C C C C C C    
832	. . . . . . . . . . . . A A A A . . A A A A A A A A    
833	. . . . . . . . . . . . C C C C . . C C C C C C C C    
834	. . . . . . . . . . . . T T T T . . T T T T T T T T    
835	. . . . . . . . . . . . A A A A . . A A A A A A A A    
836	. . . . . . . . . . . . G G G G . . G G G G G G G G    
837	. . . . . . . . . . . . A A A A . . A A A A A A A A    
838	. . . . . . . . . . . . C C C C . . C C C C C C C C    
839	. . . . . . . . . . . . A A A A . . A A A A A A A A    
840	. . . . . . . . . . . . C C C C . . C C C C C C C C    
841	. . . . . . . . . . . . G G G G . . G G G G G G G G    
842	. . . . . . . . . . . . T T T T . . T T T T T T T T    
843	. . . . . . . . . . . . T T T T . . T T T T T T T T    
844	. . . . . . . . . . . . C C C C . . C C C C C C C C    
845	. . . . . . . . . . . . A A A A . . A A A A A A A A    
846	. . . . . . . . . . . . C C C C . . C C C C C C C C    
847	. . . . . . . . . . . . C C C C . . C C C C C C C C    
848	. . . . . . . . . . . . A A A A . . A A A A A A A A    
849	. . . . . . . . . . . . C C C C . . C C C C C C C C    
850	. . . . . . . . . . . . C C C C . . C C C C C C C C    
851	. . . . . . . . . . . . C C C C . . C C C C C C C C    
852	. . . . . . . . . . . . T T T T . T T T T T T T T T    
853	. . . . . . . . . . . . C C C C . C C C C C C C C C    
854	. . . . . . . . . . . . C C C C . C C C C C C C C C    
855	. . . . . . . . . . . . C C C C . C C C C C C C C C    
856	. . . . . . . . . . . . T T T T . T T T T T T T T T    
857	. . . . . . . . . . . . C C C C . C C C C C C C C C    
858	. . . . . . . . . . . . C C C C . C C C C C C C C C    
859	. . . . . . . . . . . . T T T T . T T T T T T T T T    
860	. . . . . . . . . . . . G G G G . G G G G G G G G G    
861	. . . . . . . . . . . . G G G G . G G G G G G G G G    
862	. . . . . . . . . . . . T T T T . T T T T T T T T T    
863	. . . . . . . . . . . . T T T T . T T T T T T T T T    
864	. . . . . . . . . . . . T T T T . T T T T T T T T T    
865	. . . . . . . . . . . . G G G G . G G G G G G G G G    
866	. . . . . . . . . . . . A A A A . A A A A A A A A A    
867	. . . . . . . . . . . . T T T T . T T T T T T T T T    
868	. . . . . . . . . . . . G G G G . G G G G G G G G G    
869	. . . . . . . . . . . . A A A A . A A A A A A A A A    
870	. . . . . . . . . . . . T T T T . T T T T T T T T T    
871	. . . . . . . . . . . . G G G G . G G G G G G G G G    
872	. . . . . . . . . . . . G G G G . G G G G G G G G G    
873	. . . . . . . . . . . . G G G G . G G G G G G G G G    
874	. . . . . . . . . . . . G G G G . G G G G G G G G G    
875	. . . . . . . . . . . . A A A A . A A A A A A A A A    
876	. . . . . . . . . . . . G G G G . G G G G G G G G G    
877	. . . . . . . . . . . . A A A A . A A A A A A A A A    
878	. . . . . . . . . . . . A A A A . A A A A A A A A A    
879	. . . . . . . . . . . . A A A A . A A A A A A A A A    
880	. . . . . . . . . . . . C C C C . C C C C C C C C C    
881	. . . . . . . . . . . . T T T T . T T T T T T T T T    
882	. . . . . . . . . . . . C C C C . C C C C C C C C C    
883	. . . . . . . . . . . . A A A A . A A A A A A A A A    
884	. . . . . . . . . . . . T T T T . T T T T T T T T T    
885	. . . . . . . . . . . . T T T T . T T T T T T T T T    
886	. . . . . . . . . . . . G G G G . G G G G G G G G G    
887	. . . . . . . . . . . . C C C C . C C C C C C C C C    
888	. . . . . . . . . . . . A A A A . A A A A A A A A A    
889	. . . . . . . . . . . . C C C C . C C C C C C C C C    
890	. . . . . . . . . . . . A A A A . A A A A A A A A A    
891	. . . . . . . . . . . . T T T T . T T T T T T T T T    
892	. . . . . . . . . . . . G G G G . G G G G G G G G G    
893	. . . . . . . . . . . . . T T T . T T T T T T T T T    
894	. . . . . . . . . . . . . C C C . C C C C C C C C C    
895	. . . . . . . . . . . . . T T T . T T T T T T T T T    
896	. . . . . . . . . . . . . C C C . C C C C C C C C C    
897	. . . . . . . . . . . . . T T T . T T T T T T T T T    
898	. . . . . . . . . . . . . T T T . T T T T T T T T T    
899	. . . . . . . . . . . . . G G G . G G G G G G G G G    
900	. . . . . . . . . . . . . T T T . T T T T T T T T T    
901	. . . . . . . . . . . . . G G G . G G G G G G G G G    
902	. . . . . . . . . . . . . G G G . G G G G G G G G G    
903	. . . . . . . . . . . . . C C C . C C C C C C C C C    
904	. . . . . . . . . . . . . A A A . A A A A A A A A A    
905	. . . . . . . . . . . . . A A A . A A A A A A A A A    
906	. . . . . . . . . . . . . A A A . A A A A A A A A A    
907	. . . . . . . . . . . . . G G G . G G G G G G G G G    
908	. . . . . . . . . . . . . A A A . A A A A A A A A A    
909	. . . . . . . . . . . . . A A A . A A A A A A A A A    
910	. . . . . . . . . . . . . G G G . G G G G G G G G G    
911	. . . . . . . . . . . . . T T T . T T T T T T T T T    
912	. . . . . . . . . . . . . A T T . T T T T T T T T T    
913	. . . . . . . . . . . . . . A A . A A A A A A A A A    
914	. . . . . . . . . . . . . . G G . G G G G G G G G G    
915	. . . . . . . . . . . . . . T T . T T T T T T T T T    
916	. . . . . . . . . . . . . . T T . T T T T T T T T T    
917	. . . . . . . . . . . . . . T T . T T T T T T T T T    
918	. . . . . . . . . . . . . . T T . T T T T T T T T T    
919	. . . . . . . . . . . . . . G G . G G G G G G G G G    
920	. . . . . . . . . . . . . . A A . A A A A A A A A A    
921	. . . . . . . . . . . . . . G G . G G G G G G G G G    
922	. . . . . . . . . . . . . . A A . A A A A A A A A A    
923	. . . . . . . . . . . . . . G G . G G G G G G G G G    
924	. . . . . . . . . . . . . . T T . T T T T T T T T T    
925	. . . . . . . . . . . . . . C C . C C C C C C C C C    
926	. . . . . . . . . . . . . . A A . A A A A A A A A A    
927	. . . . . . . . . . . . . . C C . C C C C C C C C C    
928	. . . . . . . . . . . . . . C C . C C C C C C C C C    
929	. . . . . . . . . . . . . . T T . T T T T T T T T T    
930	. . . . . . . . . . . . . . C C . C C C C C C C C C    
931	. . . . . . . . . . . . . . A A . A A A A A A A A A    
932	. . . . . . . . . . . . . . G G . G G G G G G G G G    
933	. . . . . . . . . . . . . . C C . C C C C C C C C C    
934	. . . . . . . . . . . . . . T T . T T T T T T T T T    
935	. . . . . . . . . . . . . . T T . T T T T T T T T T    
936	. . . . . . . . . . . . . . G G . G G G G G G G G G    
937	. . . . . . . . . . . . . . G G . G G G G G G G G G    
938	. . . . . . . . . . . . . . G G . G G G G G G G G G    
939	. . . . . . . . . . . . . . T T . T T T T T T T T T    
940	. . . . . . . . . . . . . . T T . T T T T T T T T T    
941	. . . . . . . . . . . . . . C C . C C C C C C C C C    
942	. . . . . . . . . . . . . . C C . C C C C C C C C C    
943	. . . . . . . . . . . . . . A A A A A A A A A A A A    
944	. . . . . . . . . . . . . . A A A A A A A A A A A A    
945	. . . . . . . . . . . . . . A A A A A A A A A A A A    
946	. . . . . . . . . . . . . . G G G G G G G G G G G G    
947	. . . . . . . . . . . . . . G G G G G G G G G G G G    
948	. . . . . . . . . . . . . . G G G G G G G G G G G G    
949	. . . . . . . . . . . . . . T T T T T T T T T T T T    
950	. . . . . . . . . . . . . . G G G G G G G G G G G G    
951	. . . . . . . . . . . . . . T T T T T T T T T T T T    
952	. . . . . . . . . . . . . . A A A A A A A A A A A A    
953	. . . . . . . . . . . . . . G G G G G G G G G G G G    
954	. . . . . . . . . . . . . . T T T T T T T T T T T T    
955	. . . . . . . . . . . . . . A A A A A A A A A A A A    
956	. . . . . . . . . . . . . . T T T T T T T T T T T T    
957	. . . . . . . . . . . . . . C C C C C C C C C C C C    
958	. . . . . . . . . . . . . . A A A A A A A A A A A A    
959	. . . . . . . . . . . . . . G G G G G G G G G G G G    
960	. . . . . . . . . . . . . . C C C C C C C C C C C C    
961	. . . . . . . . . . . . . . G G G G G G G G G G G G    
962	. . . . . . . . . . . . . . T T T T T T T T T T T T    
963	. . . . . . . . . . . . . . T T T T T T T T T T T T    
964	. . . . . . . . . . . . . . T T T T T T T T T T T T    
965	. . . . . . . . . . . . . . T T T T T T T T T T T T    
966	. . . . . . . . . . . . . . T T T T T T T T T T T T    
967	. . . . . . . . . . . . . . A A A A A A A A A A A A    
968	. . . . . . . . . . . . . . A A A A A A A A A A A A    
969	. . . . . . . . . . . . . . A A A A A A A A A A A A    
970	. . . . . . . . . . . . . . G G G G G G G G G G G G    
971	. . . . . . . . . . . . . . G G G G G G G G G G G G    
972	. . . . . . . . . . . . . . C C C C C C C C C C C C    
973	. . . . . . . . . . . . . . A A A A A A A A A A A A    
974	. . . . . . . . . . . . . . A A A A A A A A A A A A    
975	. . . . . . . . . . . . . . T T T T T T T T T T T T    
976	. . . . . . . . . . . . . . C C C C C C C C C C C C    
977	. . . . . . . . . . . . . . C C C C C C C C C C C C    
978	. . . . . . . . . . . . . . C C C C C C C C C C C C    
979	. . . . . . . . . . . . . . A A A A A A A A A A A A    
980	. . . . . . . . . . . . . . G G G G G G G G G G G G    
981	. . . . . . . . . . . . . . C C C C C C C C C C C C    
982	. . . . . . . . . . . . . . A A A A A A A A A A A A    
983	. . . . . . . . . . . . . . T T T T T T T T T T T T    
984	. . . . . . . . . . . . . . T T T T T T T T T T T T    
985	. . . . . . . . . . . . . . G G G G G G G G G G G G    
986	. . . . . . . . . . . . . . T T T T T T T T T T T T    
987	. . . . . . . . . . . . . . T T T T T T T T T T T T    
988	. . . . . . . . . . . . . . A A A A A A A A A A A A    
989	. . . . . . . . . . . . . . A A A A A A A A A A A A    
990	. . . . . . . . . . . . . . T T T T T T T T T T T T    
991	. . . . . . . . . . . . . . G G G G G G G G G G G G    
992	. . . . . . . . . . . . . . A A A A A A A A A A A A    
993	. . . . . . . . . . . . . . G G G G G G G G G G G G    
994	. . . . . . . . . . . . . . C C C C C C C C C C C C    
995	. . . . . . . . . . . . . . A A A A A A A A A A A A    
996	. . . . . . . . . . . . . . C C C C C C C C C C C C    
997	. . . . . . . . . . . . . . A A A A A A A A A A A A    
998	. . . . . . . . . . . . . . T T T T T T T T T T T T    
999	. . . . . . . . . . . . . . C C C C C C C C C C C C    
1000	. . . . . . . . . . . . . . A A A A A A A A A A A A    
1001	. . . . . . . . . . . . . . A A A A A A A A A A A A    
1002	. . . . . . . . . . . . . . G G G G G G G G G G G G    
1003	. . . . . . . . . . . . . . G G G G G G G G G G G G    
1004	. . . . . . . . . . . . . . - - G - - - - - - - - -    
1005	. . . . . . . . . . . . . . C C C C C C C C C C C C    
1006	. . . . . . . . . . . . . . C C C C C C C C C C C C    
1007	. . . . . . . . . . . . . . A A A A A A A A A A A A    
1008	. . . . . . . . . . . . . . C C C C C C C C C C C C    
1009	. . . . . . . . . . . . . . C C C C C C C C C C C C    
1010	. . . . . . . . . . . . . . A A A A A A A A A A A A    
1011	. . . . . . . . . . . . . . T T T T T T T T T T T T    
1012	. . . . . . . . . . . . . . A A A A A A A A A A A A    
1013	. . . . . . . . . . . . . . T T T T T T T T T T T T    
1014	. . . . . . . . . . . . . . T T T T T T T T T T T T    
1015	. . . . . . . . . . . . . . T T T T T T T T T T T T    
1016	. . . . . . . . . . . . . . C C C C C C C C C C C C    
1017	. . . . . . . . . . . . . . T T T T T T T T T T T T    
1018	. . . . . . . . . . . . . . C C C C C C C C C C C C    
1019	. . . . . . . . . . . . . . A A A A A A A A A A A A    
1020	. . . . . . . . . . . . . . T T T T T T T T T T T T    
1021	. . . . . . . . . . . . . . T T T T T T T T T T T T    
1022	. . . . . . . . . . . . . . G G G G G G G G G G G G    
1023	. . . . . . . . . . . . . . A A A A A A A A A A A A    
1024	. . . . . . . . . . . . . . A A A A A A A A A A A A    
1025	. . . . . . . . . . . . . . G G G G G G G G G G G G    
1026	. . . . . . . . . . . . . . A A A A A A A A A A A A    
1027	. . . . . . . . . . . . . . A A A A A A A A A A A A    
1028	. . . . . . . . . . . . . . A A A A A A A A A A A A    
1029	. . . . . . . . . . . . . . T T T T T T T T T T T T    
1030	. . . . . . . . . . . . . . C C C C C C C C C C C C    
1031	. . . . . . . . . . . . . . T T T T T T T T T T T T    
1032	. . . . . . . . . . . . . . C C C C C C C C C C C C    
1033	. . . . . . . . . . . . . . G G G G G G G G G G G G    
1034	. . . . . . . . . . . . . . G G G G G G G G G G G G    
1035	. . . . . . . . . . . . . . G G G G G G G G G G G G    
1036	. . . . . . . . . . . . . . C C C C C C C C C C C C    
1037	. . . . . . . . . . . . . . C C C C C C C C C C C C    
1038	. . . . . . . . . . . . . . G G G G G G G G G G G G    
1039	. . . . . . . . . . . . . . C C C C C C C C C C C C    
1040	. . . . . . . . . . . . . . G G G G G G G G G G G G    
1041	. . . . . . . . . . . . . . C C C C C C C C C C C C    
1042	. . . . . . . . . . . . . . G G G G G G G G G G G G    
1043	. . . . . . . . . . . . . . C C C C C C C C C C C C    
1044	. . . . . . . . . . . . . . A A A A A A A A A A A A    
1045	. . . . . . . . . . . . . . C C C C C C C C C C C C    
1046	. . . . . . . . . . . . . . A A A A A A A A A A A A    
1047	. . . . . . . . . . . . . . C C C C C C C C C C C C    
1048	. . . . . . . . . . . . . . T T T T T T T T T T T T    
1049	. . . . . . . . . . . . . . T T T T T T T T T T T T    
1050	. . . . . . . . . . . . . . T T T T T T T T T T T T    
1051	. . . . . . . . . . . . . . C C C C C C C C C C C C    
1052	. . . . . . . . . . . . . . T T T T T T T T T T T T    
1053	. . . . . . . . . . . . . . G G G G G G G G G G G G    
1054	. . . . . . . . . . . . . . G G G G G G G G G G G G    
1055	. . . . . . . . . . . . . . G G G G G G G G G G G G    
1056	. . . . . . . . . . . . . . T T T T T T T T T T T T    
1057	. . . . . . . . . . . . . . C C C C C C C C C C C C    
1058	. . . . . . . . . . . . . . G G G G G G G G G G G G    
1059	. . . . . . . . . . . . . . T T T T T T T T T T T T    
1060	. . . . . . . . . . . . . . T T T T T T T T T T T T    
1061	. . . . . . . . . . . . . . G G G G G G G G G G G G    
1062	. . . . . . . . . . . . . . A A A A A A A A A A A A    
1063	. . . . . . . . . . . . . . T T T T T T T T T T T T    
1064	. . . . . . . . . . . . . . G G G G G G G G G G G G    
1065	. . . . . . . . . . . . . . T T T T T T T T T T T T    
1066	. . . . . . . . . . . . . . C C C C C C C C C C C C    
1067	. . . . . . . . . . . . . . A A A A A A A A A A A A    
1068	. . . . . . . . . . . . . . A A A A A A A A A A A A    
1069	. . . . . . . . . . . . . . G G G G G G G G G G G G    
1070	. . . . . . . . . . . . . . C C C C C C C C C C C C    
1071	. . . . . . . . . . . . . . T T T T T T T T T T T T    
1072	. . . . . . . . . . . . . . G G G G G G G G G G G G    
1073	. . . . . . . . . . . . . . T T T T T T T T T T T T    
1074	. . . . . . . . . . . . . . T T T T T T T T T T T T    
1075	. . . . . . . . . . . . . . G G G G G G G G G G G G    
1076	. . . . . . . . . . . . . . G G G G G G G G G G G G    
1077	. . . . . . . . . . . . . . A A A A A A A A A A A A    
1078	. . . . . . . . . . . . . . A A A A A A A A A A A A    
1079	. . . . . . . . . . . . . . G G G G G G G G G G G G    
1080	. . . . . . . . . . . . . . A A A A A A A A A A A A    
1081	. . . . . . . . . . . . . . G G G G G G G G G G G G    
1082	. . . . . . . . . . . . . . G G G G G G G G G G G G    
1083	. . . . . . . . . . . . . . G G G G G G G G G G G G    
1084	. . . . . . . . . . . . . . G G G G G G G G G G G G    
1085	. . . . . . . . . . . . . . T T T T T T T T T T T T    
1086	. . . . . . . . . . . . . . T T T T T T T T T T T T    
1087	. . . . . . . . . . . . . . T T T T T T T T T T T T    
1088	. . . . . . . . . . . . . . C C C C C C C C C C C C    
1089	. . . . . . . . . . . . . . A A A A A A A A A A A A    
1090	. . . . . . . . . . . . . . G G G G G G G G G G G G    
1091	. . . . . . . . . . . . . . C C C C C C C C C C C C    
1092	. . . . . . . . . . . . . . C C C C C C C C C C C C    
1093	. . . . . . . . . . . . . . C C C C C C C C C C C C    
1094	. . . . . . . . . . . . . . T T T T T T T T T T T T    
1095	. . . . . . . . . . . . . . T T T T T T T T T T T T    
1096	. . . . . . . . . . . . . . C C C C C C C C C C C C    
1097	. . . . . . . . . . . . . . A A A A A A A A A A A A    
1098	. . . . . . . . . . . . . . G G G G G G G G G G G G    
1099	. . . . . . . . . . . . . . A A A A A A A A A A A A    
1100	. . . . . . . . . . . . . . A A A A A A A A A A A A    
1101	. . . . . . . . . . . . . . T T T T T T T T T T T T    
1102	. . . . . . . . . . . . . . T T T T T T T T T T T T    
1103	. . . . . . . . . . . . . . C C C C C C C C C C C C    
1104	. . . . . . . . . . . . . . A A A A A A A A A A A A    
1105	. . . . . . . . . . . . . . G G G G G G G G G G G G    
1106	. . . . . . . . . . . . . . G G G G G G G G G G G G    
1107	. . . . . . . . . . . . . . C C C C C C C C C C C C    
1108	. . . . . . . . . . . . . . T T T T T T T T T T T T    
1109	. . . . . . . . . . . . . . C C C C C C C C C C C C    
1110	. . . . . . . . . . . . . . T T T T T T T T T T T T    
1111	. . . . . . . . . . . . . . C C C C C C C C C C C C    
1112	. . . . . . . . . . . . . . C C C C C C C C C C C C    
1113	. . . . . . . . . . . . . . A A A A A A A A A A A A    
1114	. . . . . . . . . . . . . . C C C C C C C C C C C C    
1115	. . . . . . . . . . . . . . A A A A A A A A A A A A    
1116	. . . . . . . . . . . . . . G G G G G G G G G G G G    
1117	. . . . . . . . . . . . . . C C C C C C C C C C C C    
1118	. . . . . . . . . . . . . . C C C C C C C C C C C C    
1119	. . . . . . . . . . . . . . G G G G G G G G G G G G    
1120	. . . . . . . . . . . . . . C C C C C C C C C C C C    
1121	. . . . . . . . . . . . . . T T T T T T T T T T T T    
1122	. . . . . . . . . . . . . . G G G G G G G G G G G G    
1123	. . . . . . . . . . . . . . T T T T T T T T T T T T    
1124	. . . . . . . . . . . . . . T T T T T T T T T T T T    
1125	. . . . . . . . . . . . . . C C C C C C C C C C C C    
1126	. . . . . . . . . . . . . . C C C C C C C C C C C C    
1127	. . . . . . . . . . . . . . A A A A A A A A A A A A    
1128	. . . . . . . . . . . . . . C C C C C C C C C C C C    
1129	. . . . . . . . . . . . . . G G G G G G G G G G G G    
1130	. . . . . . . . . . . . . . G G G G G G G G G G G G    
1131	. . . . . . . . . . . . . . C C C C C C C C C C C C    
1132	. . . . . . . . . . . . . . C C C C C C C C C C C C    
1133	. . . . . . . . . . . . . . A A A A A A A A A A A A    
1134	. . . . . . . . . . . . . . G G G G G G G G G G G G    
1135	. . . . . . . . . . . . . . C C C C C C C C C C C C    
1136	. . . . . . . . . . . . . . A A A A A A A A A A A A    
1137	. . . . . . . . . . . . . . T T T T T T T T T T T T    
1138	. . . . . . . . . . . . . . C C C C C C C C C C C C    
1139	. . . . . . . . . . . . . . C C C C C C C C C C C C    
1140	. . . . . . . . . . . . . . C C C C C C C C C C C C    
1141	. . . . . . . . . . . . . . G G G G G G G G G G G G    
1142	. . . . . . . . . . . . . . G G G G G G G G G G G G    
1143	. . . . . . . . . . . . . . C C C C C C C C C C C C    
1144	. . . . . . . . . . . . . . T T T T T T T T T T T T    
1145	. . . . . . . . . . . . . . A A A A A A A A A A A A    
1146	. . . . . . . . . . . . . . C C C C C C C C C C C C    
1147	. . . . . . . . . . . . . . T T T T T T T T T T T T    
1148	. . . . . . . . . . . . . . G G G G G G G G G G G G    
1149	. . . . . . . . . . . . . . A A A A A A A A A A A A    
1150	. . . . . . . . . . . . . . G G G G G G G G G G G G    
1151	. . . . . . . . . . . . . . G G G G G G G G G G G G    
1152	. . . . . . . . . . . . . . A A A A A A A A A A A A    
1153	. . . . . . . . . . . . . . A A A A A A A A A A A A    
1154	. . . . . . . . . . . . . . A A A A A A A A A A A A    
1155	. . . . . . . . . . . . . . A A A A A A A A A A A A    
1156	. . . . . . . . . . . . . . C C C C C C C C C C C C    
1157	. . . . . . . . . . . . . . A A A A A A A A A A A A    
1158	. . . . . . . . . . . . . . T T T T T T T T T T T T    
1159	. . . . . . . . . . . . . . C C C C C C C C C C C C    
1160	. . . . . . . . . . . . . . C C C C C C C C C C C C    
1161	. . . . . . . . . . . . . . C C C C C C C C C C C C    
1162	. . . . . . . . . . . . . . C C C C C C C C C C C C    
1163	. . . . . . . . . . . . . . A A A A A A A A A A A A    
1164	. . . . . . . . . . . . . . T T T T T T T T T G T T    
1165	. . . . . . . . . . . . . . T T T T T T T T T C T T    
1166	. . . . . . . . . . . . . . G G G G G G G G G G G G    
1167	. . . . . . . . . . . . . . A A A A A A A A A A A A    
1168	. . . . . . . . . . . . . . A A A A A A A A A A A A    
1169	. . . . . . . . . . . . . . T T T T T T T T T T T T    
1170	. . . . . . . . . . . . . . T T T T T T T T T T T T    
1171	. . . . . . . . . . . . . . G G G G G G G G G G G G    
1172	. . . . . . . . . . . . . . C C C C C C C C C C C C    
1173	. . . . . . . . . . . . . . T T T T T T T T T T T T    
1174	. . . . . . . . . . . . . . T T T T T T T T T T T T    
1175	. . . . . . . . . . . . . . G G G G G G G G G G G G    
1176	. . . . . . . . . . . . . . C C C C C C C C C C C C    
1177	. . . . . . . . . . . . . . A A A A A A A A A A A A    
1178	. . . . . . . . . . . . . . A A A A A A A A A A A A    
1179	. . . . . . . . . . . . . . A A A A A A A A A A A A    
1180	. . . . . . . . . . . . . . G G G G G G G G G G G G    
1181	. . . . . . . . . . . . . . C C C C C C C C C C C C    
1182	. . . . . . . . . . . . . . G G G G G G G G G G G G    
1183	. . . . . . . . . . . . . . A A A A A A A A A A A A    
1184	. . . . . . . . . . . . . . C C C C C C C C C C C C    
1185	. . . . . . . . . . . . . . C C C C C C C C C C C C    
1186	. . . . . . . . . . . . . . G G G G G G G G G G G G    
1187	. . . . . . . . . . . . . . C C C C C C C C C C C C    
1188	. . . . . . . . . . . . . . A A A A A A A A A A A A    
1189	. . . . . . . . . . . . . . C C C C C C C C C C C C    
1190	. . . . . . . . . . . . . . A A A A A A A A A A A A    
1191	. . . . . . . . . . . . . . A A A A A A A A A A A A    
1192	. . . . . . . . . . . . . . T T T T T T T T T T T T    
1193	. . . . . . . . . . . . . . T T T T T T T T T T T T    
1194	. . . . . . . . . . . . . . G G G G G G G G G G G G    
1195	. . . . . . . . . . . . . . C C C C C C C C C C C C    
1196	. . . . . . . . . . . . . . A A A A A A A A A A A A    
1197	. . . . . . . . . . . . . . A A A A A A A A A A A A    
1198	. . . . . . . . . . . . . . A A A A A A A A A A A A    
1199	. . . . . . . . . . . . . . T T T T T T T T T T T T    
1200	. . . . . . . . . . . . . . C C C C C C C C C C C C    
1201	. . . . . . . . . . . . . . C C C C C C C C C C C C    
1202	. . . . . . . . . . . . . . A A A A A A A A A A A A    
1203	. . . . . . . . . . . . . . A A A A A A A A A A A A    
1204	. . . . . . . . . . . . . . T T T T T T T T T T T T    
1205	. . . . . . . . . . . . . . C C C C C C C C C C C C    
1206	. . . . . . . . . . . . . . C C C C C C C C C C C C    
1207	. . . . . . . . . . . . . . C C C C C C C C C C C C    
1208	. . . . . . . . . . . . . . C C C C C C C C C C C C    
1209	. . . . . . . . . . . . . . T T T T T T T T T T T T    
1210	. . . . . . . . . . . . . . T T T T T T T T T T T T    
1211	. . . . . . . . . . . . . . A A A A A A A A A A A A    
1212	. . . . . . . . . . . . . . T T T T T T T T T T T T    
1213	. . . . . . . . . . . . . . T T T T T T T T T T T T    
1214	. . . . . . . . . . . . . . G G G G G G G G G G G G    
1215	. . . . . . . . . . . . . . G G G G G G G G G G G G    
1216	. . . . . . . . . . . . . . A A A A A A A A A A A A    
1217	. . . . . . . . . . . . . . A A A A A A A A A A A A    
1218	. . . . . . . . . . . . . . C C C C C C C C C C C C    
1219	. . . . . . . . . . . . . . C C C C C C C C C C C C    
1220	. . . . . . . . . . . . . . A A A A A A A A A A A A    
1221	. . . . . . . . . . . . . . G G G G G G G G G G G G    
1222	. . . . . . . . . . . . . . T T T T T T T T T T T T    
1223	. . . . . . . . . . . . . . C C C C C C C C C C C C    
1224	. . . . . . . . . . . . . . A A A A A A A A A A A A    
1225	. . . . . . . . . . . . . . C C C C C C C C C C C C    
1226	. . . . . . . . . . . . . . C C C C C C C C C C C C    
1227	. . . . . . . . . . . . . . A A A A A A A A A A A A    
1228	. . . . . . . . . . . . . . G G G G G G G G G G G G    
1229	. . . . . . . . . . . . . . T T T T T T T T T T T T    
1230	. . . . . . . . . . . . . . G G G G G G G G G G G G    
1231	. . . . . . . . . . . . . . C C C C C C C C C C C C    
1232	. . . . . . . . . . . . . . A A A A A A A A A A A A    
1233	. . . . . . . . . . . . . . A A A A A A A A A A A A    
1234	. . . . . . . . . . . . . . C C C C C C C C C C C C    
1235	. . . . . . . . . . . . . . T T T T T T T T T T T T    
1236	. . . . . . . . . . . . . . T T T T T T T T T T T T    
1237	. . . . . . . . . . . . . . T T T T T T T T T T T T    
1238	. . . . . . . . . . . . . . T T T T T T T T T T T T    
1239	. . . . . . . . . . . . . . G G G G G G G G G G G G    
1240	. . . . . . . . . . . . . . G G G G G G G G G G G G    
1241	. . . . . . . . . . . . . . C C C C C C C C C C C C    
1242	. . . . . . . . . . . . . . A A A A A A A A A A A A    
1243	. . . . . . . . . . . . . . T T T T T T T T T T T T    
1244	. . . . . . . . . . . . . . A A A A A A A A A A A A    
1245	. . . . . . . . . . . . . . . C C C C C C C C C C C    
1246	. . . . . . . . . . . . . . . T T T T T T T T T T T    
1247	. . . . . . . . . . . . . . . T T T T T T T T T T T    
1248	. . . . . . . . . . . . . . . T T T T T T T T T T T    
1249	. . . . . . . . . . . . . . . A A A A A A A A A A A    
1250	. . . . . . . . . . . . . . . C C C C C C C C C C C    
1251	. . . . . . . . . . . . . . . A A A A A A A A A A A    
1252	. . . . . . . . . . . . . . . G G G G G G G G G G G    
1253	. . . . . . . . . . . . . . . T T T T T T T T T T T    
1254	. . . . . . . . . . . . . . . G G G G G G G G G G G    
1255	. . . . . . . . . . . . . . . G G G G G G G G G G G    
1256	. . . . . . . . . . . . . . . G G G G G G G G G G G    
1257	. . . . . . . . . . . . . . . G G G G G G G G G G G    
1258	. . . . . . . . . . . . . . . G G G G G G G G G G G    
1259	. . . . . . . . . . . . . . . T T T T T T T T T T T    
1260	. . . . . . . . . . . . . . . A A A A A A A A A A A    
1261	. . . . . . . . . . . . . . . C C C C C C C C C C C    
1262	. . . . . . . . . . . . . . . T T T T T T T T T T T    
1263	. . . . . . . . . . . . . . . C C C C C C C C C C C    
1264	. . . . . . . . . . . . . . . T T T T T T T T T T T    
1265	. . . . . . . . . . . . . . . T T T T T T T T T T T    
1266	. . . . . . . . . . . . . . . G G G G G G G G G G G    
1267	. . . . . . . . . . . . . . . A A A A A A A A A A A    
1268	. . . . . . . . . . . . . . . C C C C C C C C C C C    
1269	. . . . . . . . . . . . . . . G G G G G G G G G G G    
1270	. . . . . . . . . . . . . . . T T T T T T T T T T T    
1271	. . . . . . . . . . . . . . . G G G G G G G G G G G    
1272	. . . . . . . . . . . . . . . T T T T T T T T T T T    
1273	. . . . . . . . . . . . . . . C C C C C C C C C C C    
1274	. . . . . . . . . . . . . . . G G G G G G G G G G G    
1275	. . . . . . . . . . . . . . . A A A A A A A A A A A    
1276	. . . . . . . . . . . . . . . A A A A A A A A A A A    
1277	. . . . . . . . . . . . . . . C C C C C C C C C C C    
1278	. . . . . . . . . . . . . . . A A A A A A A A A A A    
1279	. . . . . . . . . . . . . . . G G G G G G G G G G G    
1280	. . . . . . . . . . . . . . . T T T T T T T T T T T    
1281	. . . . . . . . . . . . . . . A A A A A A A A A A A    
1282	. . . . . . . . . . . . . . . T T T T T T T T T T T    
1283	. . . . . . . . . . . . . . . T T T T T T T T T T T    
1284	. . . . . . . . . . . . . . . G G G G G G G G G G G    
1285	. . . . . . . . . . . . . . . T T T T T T T T T T T    
1286	. . . . . . . . . . . . . . . T T T T T T T T T T T    
1287	. . . . . . . . . . . . . . . A A A A A A A A A A A    
1288	. . . . . . . . . . . . . . . C C C C C . C C C C C    
1289	. . . . . . . . . . . . . . . A A A A A . A A A A A    
1290	. . . . . . . . . . . . . . . G G G G G . G G G G G    
1291	. . . . . . . . . . . . . . . A A A A A . A A A A A    
1292	. . . . . . . . . . . . . . . A A A A A . A A A A A    
1293	. . . . . . . . . . . . . . . C C C C C . C C C C C    
1294	. . . . . . . . . . . . . . . T T T T T . T T T T T    
1295	. . . . . . . . . . . . . . . G G G G G . G G G G G    
1296	. . . . . . . . . . . . . . . G G G G G . G G G G G    
1297	. . . . . . . . . . . . . . . C C C C C . . C C C C    
1298	. . . . . . . . . . . . . . . T T T T T . . T T T T    
1299	. . . . . . . . . . . . . . . T . T T T . . T T T T    
1300	. . . . . . . . . . . . . . . C . C C C . . C C C C    
1301	. . . . . . . . . . . . . . . A . C A A . . A A A A    
1302	. . . . . . . . . . . . . . . A . . A A . . A A A A    
1303	. . . . . . . . . . . . . . . A . . A A . . A A A A    
1304	. . . . . . . . . . . . . . . C . . C C . . C C C C    
1305	. . . . . . . . . . . . . . . C . . C C . . C C C C    
1306	. . . . . . . . . . . . . . . A . . A A . . A A A A    
1307	. . . . . . . . . . . . . . . C . . C C . . C C C C    
1308	. . . . . . . . . . . . . . . T . . T T . . T T T T    
1309	. . . . . . . . . . . . . . . G . . G G . . G G G G    
1310	. . . . . . . . . . . . . . . A . . A A . . A A A A    
1311	. . . . . . . . . . . . . . . G . . G G . . G G G G    
1312	. . . . . . . . . . . . . . . G . . G G . . G G G G    
1313	. . . . . . . . . . . . . . . T . . T T . . T T T T    
1314	. . . . . . . . . . . . . . . T . . T T . . T T T T    
1315	. . . . . . . . . . . . . . . G . . G G . . G G G G    
1316	. . . . . . . . . . . . . . . A . . A A . . A A A A    
1317	. . . . . . . . . . . . . . . C . . . C . . C C C C    
1318	. . . . . . . . . . . . . . . A . . . A . . A A A A    
1319	. . . . . . . . . . . . . . . C . . . C . . C C C C    
1320	. . . . . . . . . . . . . . . G . . . G . . G G G G    
1321	. . . . . . . . . . . . . . . . . . . . . . C C C C    
1322	. . . . . . . . . . . . . . . . . . . . . . T T T T    
1323	. . . . . . . . . . . . . . . . . . . . . . T T T T    
1324	. . . . . . . . . . . . . . . . . . . . . . C C C C    
1325	. . . . . . . . . . . . . . . . . . . . . . A A A A    
1326	. . . . . . . . . . . . . . . . . . . . . . G G G G    
1327	. . . . . . . . . . . . . . . . . . . . . . G G G G    
1328	. . . . . . . . . . . . . . . . . . . . . . C C C C    
1329	. . . . . . . . . . . . . . . . . . . . . . T T T T    
1330	. . . . . . . . . . . . . . . . . . . . . . A A A A    
1331	. . . . . . . . . . . . . . . . . . . . . . C C C C    
1332	. . . . . . . . . . . . . . . . . . . . . . T T T T    
1333	. . . . . . . . . . . . . . . . . . . . . . T T T T    
1334	. . . . . . . . . . . . . . . . . . . . . . G G G G    
1335	. . . . . . . . . . . . . . . . . . . . . . C C C .    
1336	. . . . . . . . . . . . . . . . . . . . . . T T T .    
1337	. . . . . . . . . . . . . . . . . . . . . . G G G .    
1338	. . . . . . . . . . . . . . . . . . . . . . G G G .    
1339	. . . . . . . . . . . . . . . . . . . . . . C C C .    
1340	. . . . . . . . . . . . . . . . . . . . . . T T T .    
1341	. . . . . . . . . . . . . . . . . . . . . . T T T .    
1342	. . . . . . . . . . . . . . . . . . . . . . C C C .    
1343	. . . . . . . . . . . . . . . . . . . . . . C C C .    
1344	. . . . . . . . . . . . . . . . . . . . . . G G G .    
1345	. . . . . . . . . . . . . . . . . . . . . . T T T .    
1346	. . . . . . . . . . . . . . . . . . . . . . A A A .    
1347	. . . . . . . . . . . . . . . . . . . . . . A A A .    
1348	. . . . . . . . . . . . . . . . . . . . . . C C . .    
1349	. . . . . . . . . . . . . . . . . . . . . . A . . .    
1350	. . . . . . . . . . . . . . . . . . . . . . T . . .    
1351	. . . . . . . . . . . . . . . . . . . . . . T . . .    
1352	. . . . . . . . . . . . . . . . . . . . . . C . . .    
1353	. . . . . . . . . . . . . . . . . . . . . . C . . .    
1354	. . . . . . . . . . . . . . . . . . . . . . C . . .    
1355	. . . . . . . . . . . . . . . . . . . . . . C . . .    
1356	. . . . . . . . . . . . . . . . . . . . . . C . . .
```
